# Orientation and Dispersion Evolution of Carbon Nanotubes in Ultra High Molecular Weight Polyethylene Composites under Extensional-Shear Coupled Flow: A Dissipative Particle Dynamics Study

**DOI:** 10.3390/polym11010154

**Published:** 2019-01-17

**Authors:** Junxia Wang, Changlin Cao, Xiaochuan Chen, Shijie Ren, Yu Chen, Dingshan Yu, Xudong Chen

**Affiliations:** 1 Key Laboratory for Polymeric Composite and Functional Materials of Ministry of Education and Key Laboratory of High Performance Polymer–based Composites of Guangdong Province, School of Chemistry, Sun Yat-Sen University, Guangzhou 510275, China; wangjx58@mail.sysu.edu.cn (J.W.); caochlin3@mail.sysu.edu.cncom (C.C.); chenxch29@mail.sysu.edu.cn (X.C.); 2 State Key Laboratory of Polymer Materials Engineering, Sichuan University, Chengdu 610065, China; rensj@scu.edu.cn; 3 Beijing Huateng Hightech Co.Ltd, Beijing 10084, China; chenyu6911@163.com

**Keywords:** orientation, dispersion, CNTs/UHMWPE composites, extensional–shear coupled flow, DPD simulation

## Abstract

The property of carbon nanotubes (CNTs)-based composites are significantly dependent on the orientation and dispersion evolution of CNTs in the polymer matrix. In this work, the dissipative particle dynamics (DPD) simulations are employed to discover the orientation and dispersion evolution of CNTs in ultra–high molecular weight polyethylene (UHMWPE) under extensional–shear coupled flow conditions for the first time. In this paper, we investigate the roles of the increasing extensional-shear coupled rate in morphology of CNTs/UHMWPE composites by varying CNTs concentration and observe that the system under consideration lies in the same evolution morphologies. When comparing our results for various morphologies, we notice that the orientation is affected more significantly by changing the extensional-shear coupled rates. A good alignment appears with an increase of extensional-shear coupled rates, which transform it into ordered morphology. In addition, a higher extensional-shear coupled rate does not necessarily contribute to better dispersion even though CNTs concentration varies, as shown by the mean square displacement (MSD) and the relative concentration distribution functions of CNTs in CNTs/UHMWPE composites.

## 1. Introduction 

Ultra–high molecular weight polyethylene (UHMWPE), with molecular weight numbering in the millions, is composed of extremely long polymeric chains of ethylene monomers, which exhibit superior toughness, high abrasion resistance, a low friction coefficient, low moisture uptake, and excellent chemical stability. This demonstrates a variety of technological applications ranging from bearing components and super tough engineering plastics to medical materials in total joint replacement. In spite of its excellent properties, the usage of UHMWPE in some demanding applications has been limited due to various constraints such as its low load bearing capacity, thermal instability, creep under static conditions, poor shear modulus, and strength. Consequently, carbon nanotube (CNTs)–reinforced UHMWPE composites caught the attention of researchers in recent years and were subjected to many experimental studies reporting the reinforcing effects of CNTs on UHMWPE, i.e., the significant improvement in the wear resistance [1,2], tribological behavior [3,4], electrical performance [5,6], mechanical properties [7,8], thermal stability [9,10], and antioxidant resistance [11,12]. 

Due to the enhanced mechanical, thermal, and electrical properties of CNTs-polymer composites, many experimental and theoretical studies have been pursued and are still going on. Moreover, simulation methods have been performed extensively to investigate the structure and dynamics of CNTs-polymer composites ranging from large-scale continuum-mechanics approaches, i.e., finite element analysis, down to atomistic simulation in which the most common is the molecular dynamics (MD) method. Recently, dissipative particle dynamics (DPD) techniques have found increasing application for simulating the behavior of CNTs systems because of the larger time and length scales possible than the MD method, such as the adsorption of the surfactants on the CNTs surface [13] or inside the CNTs [14], the interaction parameters between CNTs and water [15], and the morphology and dynamics of CNTs-polymer composites [16,17]. The property of CNTs-based composites are significantly dependent on the orientation and dispersion evolution of CNTs in the polymer matrix. The dispersion of CNTs into a polymer matrix has already been modeled successfully using DPD and Flory-huggins theory [18] in which the CNTs are modeled as simple chains with certain rigidity, discarding the tubular structure of CNTs. Moreover, DPD is a satisfactory method to tackle problems associated with complex flow structures [19]. Vo et al. [20] performed DPD simulations to study the conformation of polyvinyl pyrrolidone (PVP) grafted on CNTs and the physical adsorption of PVP under shear flow. It is concluded that the polymer could be in one of three configurations: adsorbed, shear-affected, and separated, depending on the shear rate. Zhou et al. [21] investigated the dispersion and alignment of CNTs in epoxy resin composites in equilibrated and shear flow conditions using the DPD method and found that CNTs tend to orient to the flow direction. Notable improvement of the alignment is achieved by increasing the volume fraction and the length of CNTs. 

Ever since a vane extruder was invented by Qu et al. [22,23,24], it has been widely applied in industrial production of UHMWPE-based materials, as well as CNTs/UHMWPE, due to the distinct advantage of efficient positive displacement conveying and distributive mixing. Homogeneous dispersion of CNTs in UHMWPE has been successfully realized without the aid of any additive or solvents, where dried UHMWPE powder and CNTs were manually premixed by tumbling in a plastic zip-lock bag [25]. Actually, in the extrusion process, the composite melts undergo a combined extensional deformation and shear deformation. However, to the best of our knowledge, understanding the behavior of CNTs/UHMWPE composites in response to extensional-shear coupled flow field by simulation is still scarce, except for our earlier effort [26] in which the effect of varying extensional-shear coupled loading on the deformation and stress response of CNTs/UHMWPE composites was investigated using finite element numerical simulation. Using the finite element method, the dynamic behavior can be analyzed at the largest scales, but it completely discards the atomic structure of the CNTs and UHMWPE, which relies only on their macroscopic elastic parameters. 

For CNTs-based composites, if these materials are to be utilized as effective reinforcements in polymer composites, good orientation and proper dispersion of CNTs in a polymer matrix have to be guaranteed since CNTs are easy to agglomerate and entangle due to their size and high aspect ratio. Herein, with respect to the long-chain system, the DPD method is needed to analyze the orientation and dispersion transition of CNTs in the UHMWPE associated with extensional-shear coupled flow field, which was not reported before.

## 2. Simulation Details

**Figure 1:**
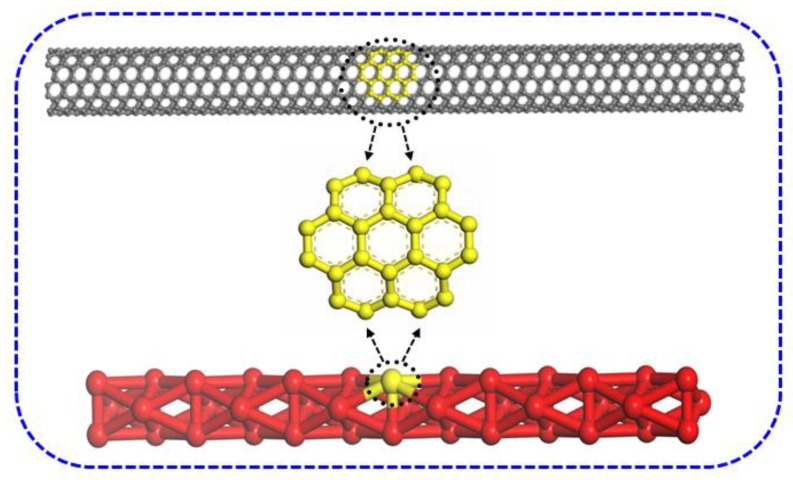
The coarse-grained model of SWCNTs (6,6).

The coarse-grained model of single-walled carbon nanotubes (SWCNTs) (6,6) with a diameter of 8.14 Å and length of 103 Å is displayed in Figure 1, where 24 carbons are grouped as one particle. Every UHMWPE chain consists of 13,914 beads. The box contains 24,000 beads and the number density is kept as 3. The SWCNTs particle is stationary and treated as a rigid body. To incorporate rigidity in the bending of the nanotube, the bond length is taken as 0.75 DPD unit and the bond constant as 20 k_B_T for CNTs. The angle and angle constant for CNTs are taken as 180° and 40 k_B_T, respectively. The volume of the CNTs particle is about 480Å^3^. The solubility parameter of UHMWPE and SWCNTs is 16.50 and 18.69 (J/cm^3^)^0.5^, which is obtained from References [27,28], respectively. All the simulations were carried out for 1 × 10^5^ steps with a time step of 1 fs in reduced DPD units.

For a combination of shear flow and extensional flow, the velocity gradient ∇u is given by Equation (1) [29] below.

(1) $$\nabla u=\left(\begin{array}{ccc} -\dot{\varepsilon } & \dot{\gamma } & 0\\ \dot{\gamma } & -\dot{\varepsilon } & 0\\ 0 & 0 & 2\dot{\varepsilon } \end{array}\right)$$

where $\dot{\gamma }$ is the shear rate and $\dot{\varepsilon }$ is the extensional rate.

As proposed in our previous study [23], in DPD simulation, pressure can be imposed on each bead to drive the flow because the normal pressure is in proportion to the force and simple extensional flow is generated using the Souza-Martins method. Consequently, in this study, referring to the velocity gradient ∇u, the extensional-shear coupled flow can be described as Equation (2) in which the equivalent hydrostatic pressure is zero. The quantitative relationship between pressure *P* (GPa) and the shear rate $\dot{\gamma }$ or extensional rate $\dot{\varepsilon }$ is still unkown.

(2) $$P_{E-S}=\left(\begin{array}{ccc} P_{xx} & P_{xy} & P_{xz}\\ P_{yx} & P_{yy} & P_{yz}\\ P_{zx} & P_{zy} & P_{zz} \end{array}\right)=\left(\begin{array}{ccc} P_{xx} & P_{xy} & 0\\ P_{yx} & P_{xx} & 0\\ 0 & 0 & -2P_{xx} \end{array}\right)$$

## 3. Results and Discussion

**Figure 2:**
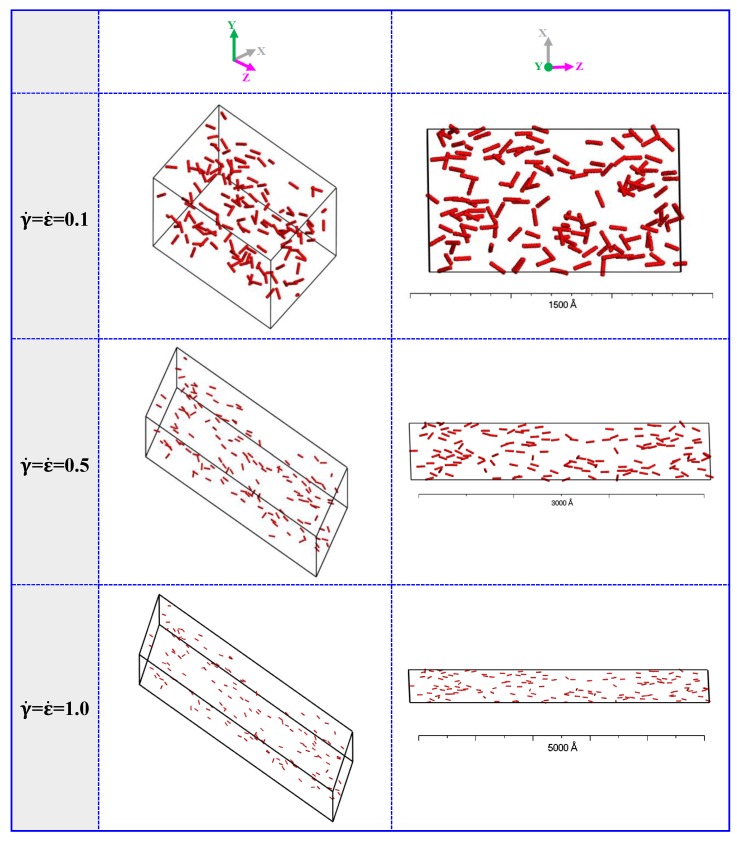
The structure of CNTs/UHMWPE composites at 10% CNTs in response to different extensional-shear coupled rates. The beads of PE are not displayed in this study and beads of CNTs are displayed in CPK style with a CPK scale of 2.5 for clarity.

In this simulation, the Souza-Martins method is performed for driving the extensional-shear coupled flow field, by combining the simple extensional flow field and the simple shear flow field. To describe the orientation and dispersion evolution of CNTs (10%) in UHMWPE under extensional-shear coupled flow, morphological structure evolution under a low, moderate, and high extensional-shear coupled rates (0.1, 0.5 and 1.0) are calculated, where the extensional-shear coupled rate is composed of shear rate $\dot{\gamma }$ and extensional rate $\dot{\varepsilon }$, $\dot{\varepsilon }$ = $\dot{\gamma }$ in the current paper. The typical simulation results are illustrated in Figure 2. To observe the CNTs morphology clearly, beads of PE are not displayed in this study and beads of CNTs are displayed in CPK style with CPK scale of 2.5 (CPK style indicates that atoms are rendered as spheres with radii that are related to the radii of the beads). These results confirm that the final states of the orientation is highly dependent on the extensional-shear coupled rate. When at a low coupled rate (i.e., 0.1), CNTs tend to form disordered morphology. By increasing the coupled rate, CNTs tend to align along the elongated direction. In the case of a high coupled rate (1.0), the ordering alignment is noticeable. However, an increment of extensional-shear coupled rate yields less effect on the dispersion of CNTs in UHMWPE. This means that the higher extensional-shear coupled rate does not necessarily contribute to much better dispersion.

**Figure 3:**
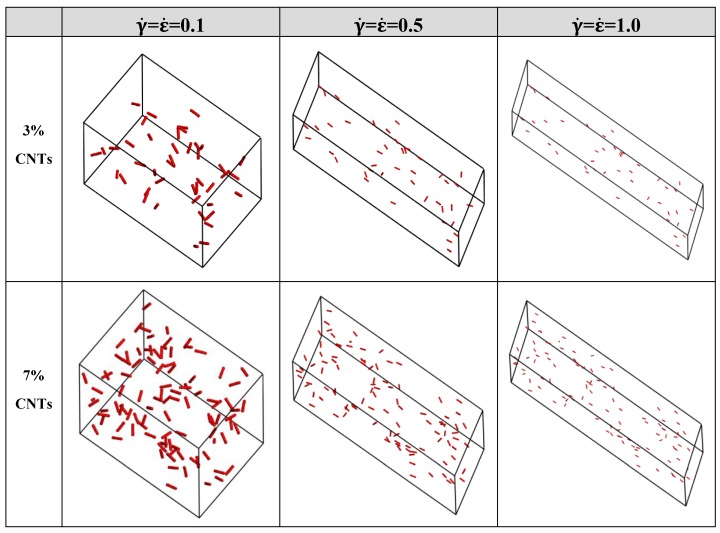
The structure of CNTs/UHMWPE composites at different CNTs mass fractions under a low, moderate, and high extensional-shear coupled rates (0.1, 0.5 and 1.0). The beads of PE are not displayed in this study and beads of CNTs are displayed in CPK style with CPK scale of 2.5 for clarity.

Compliant to proven flow facts on orientation and dispersion of CNTs in UHMWPE, mass fraction of CNTs in UHMWPE is taken into consideration. Figure 3 displays the morphologic structures of system of CNTs/UHMWPE composites at 3% and 7% CNTs under a low, moderate and high extensional-shear coupled rates (0.1, 0.5 and 1.0). A common feature between different flow is that the alignment and dispersion of CNTs in UHMWPE behaves rather similarly under different rates since CNTs concentration varies. The orientation arrangement of CNTs is improved with increasing extensional-shear coupled rates from 0.1 to 1.0, which suggests that the increase of coupled rates promotes the CNTs to transform into ordered morphology. Furthermore, the quality of the dispersion is not significantly improved by imposing a higher extensional-shear coupled rate, even though CNTs concentration varies because CNTs dispersed evenly in the UHMWPE matrix within the considered CNTs content, in accordance with experimental results by Yin et al.

**Figure 4:**
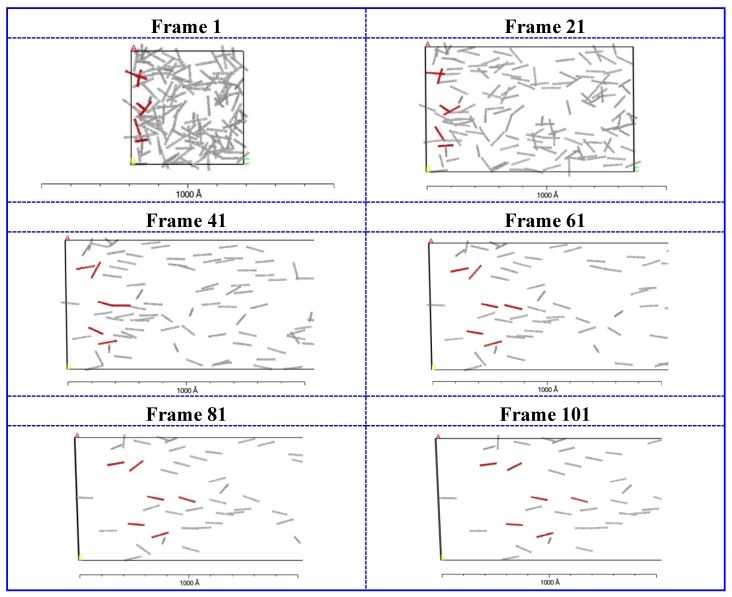
Simulation snapshots of CNTs/UHMWPE composites at 10% CNTs, obtained with a coupled rate of 1.0. The snapshots are taken every 2 × 10^4^ timesteps.

In Figure 4, the dynamic process of CNTs alignment in CNTs/UHMWPE composites at 10% CNTs under extensional-shear coupled flow field, obtained with a coupled rate of 1.0, is presented. The simulation snapshots are taken every 2 × 10^4^ timesteps. Frame 1 is the initial structure and some CNTs clusters can be found. Frame 21 shows that the CNTs begin to align along the flow direction and are not disordered absolutely. After 1 × 10^5^ timesteps, the CNTs are highly oriented and the initial disordered morphology disappears, instead of the orientation arrangement. The findings confirm that the extensional-shear coupled flow has a significant influence on the CNTs orientation. Since extensional-shear coupled flow is imposed, the system is elongated and then the CNTs are far apart and gradually align along the flow direction. Furthermore, the orientation arrangement still exists over another 2 × 10^4^ time steps (see Frame 81 and 101). Therefore, the orientation arrangement under extensional-shear coupled flow field is stable, based on Souza-Martins method. 

**Figure 5:**
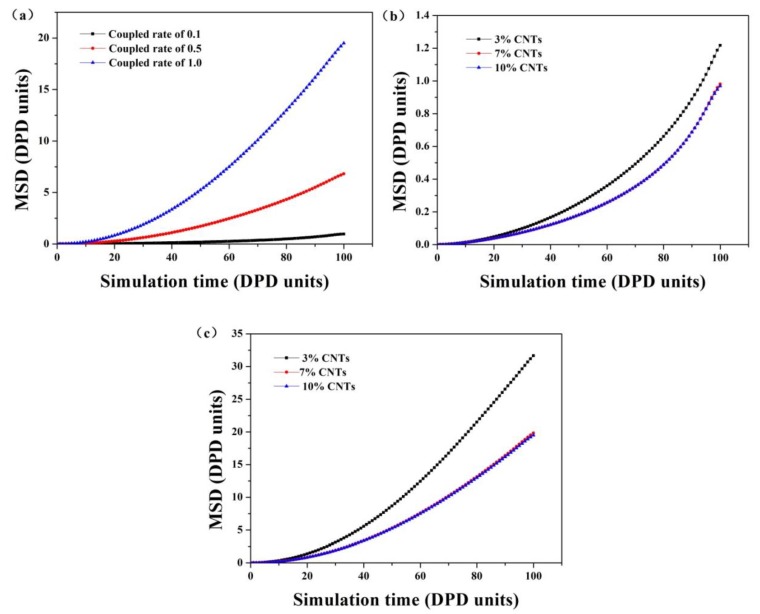
The MSD plots of CNTs in CNTs/UHMWPE composites related to: (**a**) different extensional-shear coupled rates (**b**,**c**) CNTs concentrations, with coupled rate of 0.1 and 1.0, respectively.

In addition to the morphologic structures and dynamic process of CNTs in CNTs/UHMWPE composites, the mean square displacement (MSD) and the relative concentration distribution functions of CNTs in CNTs/UHMWPE composites are evaluated to further understand the structural and dynamical properties under extensional-shear coupled flow field. Figure 5 illustrates the MSD plots of CNTs in response to different extensional-shear coupled rates and CNTs concentrations. For a given coupled rates range, it shows that the MSD of CNTs in a 10% CNTs system under coupled rate of 1.0 is much higher than that under a coupled rate of 0.1 and 0.5. This can be attributed to the CNTs alignment as extensional-shear coupled rates increase. It is also found that the change of MSD with time depends on the CNTs concentration in a nonlinear fashion, for a given time. An increase of the CNTs concentration restricts the CNTs dynamics and, therefore, reduces its diffusion. 

**Figure 6:**
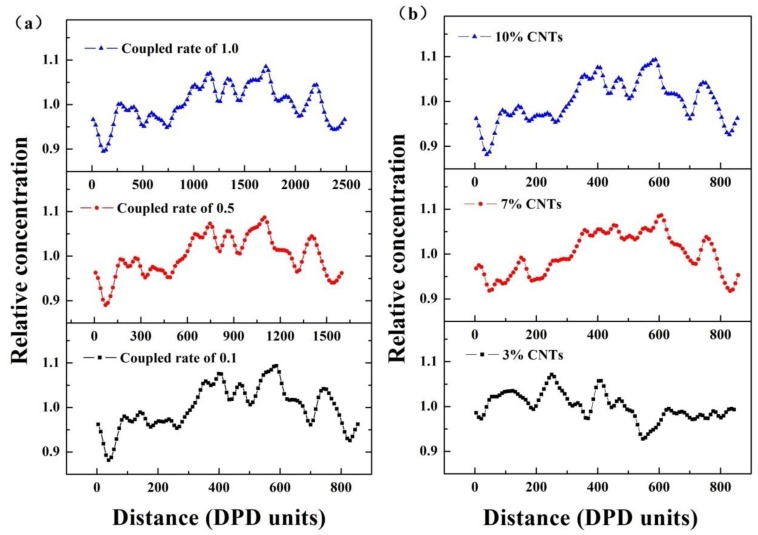
The relative concentration of CNTs in CNTs/UHMWPE composites along the Z direction (frames to average) related to: (**a**) different extensional-shear coupled rates and (**b**) CNTs concentrations.

Figure 6 demonstrates the relative concentration distribution functions of CNTs in CNTs/UHMWPE composites corresponding to the morphologies in Figure 2 and Figure 3. The relative concentration is given by the ratio of concentration of a type of bead in the slab to its average concentration across the entire system. By comparing the number and magnitude of the peak values existing in the relative concentration distribution functions, the dispersion state can be indirectly evaluated. The fewer peaks and the smaller the peaks are, more uniform the dispersion presents in CNTs/UHMWPE composites. As seen in Figure 6a, it is clear that the relative concentration evolution is similar even though extensional-shear coupled rate varies, which suggests that the higher extensional-shear coupled rate does not necessarily contribute to much better dispersion, consistent with the above results from morphologies in Figure 2 and Figure 3. Judging from Figure 6b, it can be concluded that the dispersion of CNTs in UHMWPE is slightly impacted by CNTs concentration because they are uniformly dispersed for the systems below 10% CNTs.

## 4. Conclusions

In this contribution, we focus on DPD simulation studies performed to provide a molecular-level understanding of flow-induced orientation and dispersion evolutions of CNTs in UHMWPE, subjected to extensional-shear coupled flow, for the first time. Through the morphological transition at different extensional-shear coupled rate levels, it is found that the final states of the orientation are highly dependent on the extensional-shear coupled rate and the CNTs all undergo a shift from disordered morphology to well-ordered alignment by increasing the extensional-shear coupled rate. Additionally, the dispersion of CNTs in UHMWPE is not significantly enhanced by imposing a higher extensional-shear coupled rate and is slightly impacted by the CNTs concentration. These findings agree well with those obtained from the MSD and the relative concentration distribution functions of CNTs in CNTs/UHMWPE composites. The results of this work lead to a better understanding regarding the orientation and dispersion induced by the external flow field.
